# Hearing impairment in patients with axial spondyloarthritis: association with chronic inflammatory burden and neurobiological proteins

**DOI:** 10.3389/fimmu.2026.1911654

**Published:** 2026-07-29

**Authors:** Iván Arias-de la Rosa, María Lourdes Ladehesa Pineda, Jesus Castellano-Curado, Leonardo Rodríguez-Pérez, Carlos M. Collantes-Sánchez, Carlos Pérez-Sánchez, Miriam Ruiz-Ponce, Antonio Manuel Barranco, Jesús Eduardo Martín-Salazar, Pedro Ortiz-Buitrago, María del Carmen Ábalos-Aguilera, Desiree Ruiz-Vilchez, María Ángeles Puche-Larrubia, Chary Lopez-Pedrera, Alejandro Escudero-Contreras, Eduardo Collantes-Estévez, Nuria Barbarroja, Clementina López-Medina

**Affiliations:** 1 Department of Gastroenterology, Hospital General de Tomelloso, Tomelloso, Spain/Instituto de Investigación Sanitaria de Castilla-La Mancha, Toledo, Spain; 2 Centro de Investigación Biomédica en Red de Enfermedades Hepáticas y Digestivas, Madrid, Spain; 3 Rheumatology service/Department of Medical and Surgical Sciences, Maimonides Institute for Research in Biomedicine of Cordoba (IMIBIC)/University of Cordoba/Reina Sofia University Hospital, Córdoba, Spain; 4 Orthopaedic Surgery and Traumatology Department, Servicio Andaluz de Salud (SAS), Reina Sofia University Hospital, Córdoba, Spain; 5 Department of Otorhinolaryngology, Reina Sofia University Hospital, Córdoba, Spain; 6 Cobiomic Bioscience S.L, Cordoba, Spain

**Keywords:** axial spondyloarthritis, chronic inflammatory burden, neurobiological pathways, proteomics, sensorineural hearing loss, structural damage

## Abstract

**Objective:**

To characterize hearing impairment in patients with axial spondyloarthritis (axSpA) and identify associated clinical and molecular factors.

**Methods:**

A cross-sectional study was conducted including 53 patients with axSpA from the REGISPON-3 registry and 17 age- and sex-matched healthy controls (HCs). All participants underwent pure-tone audiometry. Clinical variables included disease duration, structural damage assessed by modified Stoke Ankylosing Spondylitis Spinal Score (mSASSS), inflammatory markers, and persistent inflammation status based on serial C-reactive protein (CRP) measurements during the previous 5 years. Serum proteomic profiling focused on neurobiological pathways was performed. Multivariable regression analyses were adjusted for potential confounding factors.

**Results:**

Hearing loss was significantly more frequent in patients with axSpA compared with HCs (86.8%), predominantly bilateral sensorineural hearing loss. Patients with axSpA showed higher hearing thresholds across multiple frequencies, particularly at high frequencies. Axial SpA patients with hearing impairment were older, had longer disease duration, worse ASAS-HI scores, higher structural damage, and greater persistent inflammatory burden in comparison with those without hearing impairment. Mean CRP levels during the previous 5 years were significantly higher in patients with hearing impairment, and the prevalence of hearing loss progressively increased across inflammation persistence groups. Multivariable analyses identified higher circulating levels of HAGH, GDF8, and SPOCK1 as independently associated with hearing impairment after adjustment for structural damage, inflammation, age, sex, disease duration, and NSAID use.

**Conclusions:**

Hearing impairment is highly prevalent in axSpA and is associated with cumulative inflammation, structural damage, and an exploratory circulating neurobiological protein profile that requires validation in larger longitudinal studies.

## Introduction

Axial spondyloarthritis (axSpA) is a chronic inflammatory disease associated not only with musculoskeletal involvement but also with several extra-musculoskeletal manifestations and systemic comorbidities related to persistent inflammation (1). Chronic inflammatory burden may contribute to long-term cardiovascular, neurological, and sensory complications (1). Among these manifestations, hearing impairment, particularly sensorineural hearing loss, has increasingly been reported in patients with ankylosing spondylitis compared with healthy controls (2–4). A recent systematic review and meta-analysis further supported the association between axSpA and hearing impairment, although the available evidence remains heterogeneous regarding prevalence estimates, audiological patterns, and associated disease-related factors (5). Moreover, data specifically focused on the broader spectrum of axSpA, including non-radiographic disease, are still limited. In this regard, Yağcı et al. demonstrated that hearing loss may also occur in both radiographic and non-radiographic axSpA patients (6). The biological mechanisms underlying hearing loss in axSpA are not fully understood. Chronic systemic inflammation, immune-mediated inner ear injury, microvascular dysfunction, and neuroinflammatory pathways have all been proposed as potential contributors to cochlear and auditory pathway damage (7, 8). Previous studies evaluating cochlear function in axSpA patients have suggested that inflammatory-mediated cochlear injury may play a role in the development of sensorineural hearing loss (9, 10). However, the clinical and molecular mechanisms underlying hearing impairment in axSpA remain poorly understood. Therefore, this study aimed to characterize hearing loss in axSpA and identify associated clinical factors and potential molecular signatures through serum proteomic profiling.

## Methods

### Study design and population

We conducted a cross-sectional study including patients with axSpA from Reina Sofia University Hospital (Córdoba, Spain) participating in the REGISPON-3 registry (11), consecutively evaluated by the Otolaryngology Department. Patients with previously diagnosed hearing loss of known etiology were excluded. Healthy controls were recruited from the same institution for age- and sex-matched audiometric comparison only, HLA-B27 typing and CRP were not determined in this group. The study was approved by the Ethics Committee of Hospital Universitario Reina Sofía, and all procedures were conducted in accordance with the Declaration of Helsinki.

### Audiological assessment

All participants underwent pure-tone audiometry. Air conduction thresholds were measured at standard frequencies (125–8000 Hz), and bone conduction was assessed when clinically indicated. Hearing thresholds were recorded as the minimum intensity (in decibels, dB) required to detect sound at each frequency. Hearing loss was the primary outcome and was defined as a threshold >20 dB at any frequency. Although 20 dB corresponds to the lower limit of normal hearing established by the WHO (12), this per-frequency criterion is more sensitive than the WHO pure-tone average and was chosen to capture early, high-frequency threshold elevations.

### Clinical variables

Secondary variables included demographic characteristics, disease duration, structural damage assessed by mSASSS, inflammatory markers (c-reactive protein [CRP]), and persistent inflammation status. Persistent inflammation was retrospectively assessed using serial CRP measurements collected during the previous 5 years of routine follow-up. To ensure longitudinal representativeness, patients were required to have at least one CRP determination per year. CRP values >5 mg/L were considered indicative of active inflammation. For each patient, the proportion of CRP measurements exceeding this threshold was calculated and used to classify inflammatory burden over time. Patients were categorized as having non-persistent inflammation when ≤25% of CRP measurements were >5 mg/L, intermediate inflammation when 26–84% of measurements were >5 mg/L, and persistent inflammation when ≥85% of measurements were >5 mg/L. CRP values obtained during documented infections or other inflammatory conditions unrelated to axSpA were excluded from the analyses. This classification was intended to reflect both the frequency and consistency of systemic inflammatory activity over time (13).

### Proteomic analysis

Protein profiling was performed using validated neurology panel using proximity extension assay technology provided by Olink Proteomics (Cobiomic Bioscience (Córdoba, España), enabling the simultaneous quantification of 92 proteins directly involved in neurobiological processes (https://cobiomicbioscience.com/services/).

### Statistical analysis

Descriptive analyses were performed using means (standard deviation) or frequencies (percentages), as appropriate. Between-group comparisons were conducted using Student’s t test or Mann–Whitney U test for continuous variables, and chi-square test for categorical variables. Normality and homogeneity were assessed using the Shapiro–Wilk and Levene tests, respectively. For the comparison of hearing loss prevalence between axSpA patients and healthy controls, effect sizes were estimated using the odds ratio, risk ratio, and absolute risk difference, each with corresponding 95% confidence intervals. To evaluate the association between serum protein expression and hearing impairment, multivariable linear regression analyses were performed adjusting for potential confounding variables, including persistent inflammation status, total mSASSS, sex, disease duration, age, and NSAID use. Regression coefficients (β) with corresponding 95% confidence intervals were estimated. All tests were two-sided, with a significance level of *p* < 0.05. Statistical analyses were performed using SPSS version 25 (IBM Corp., Armonk, NY, USA) and GraphPad Prism version 9.4.1 (GraphPad Software, San Diego, CA, USA).

## Results

### Hearing loss is more frequent in patients with axSpA

Among patients with axSpA, 91.7% fulfilled the criteria for radiographic axSpA (r-axSpA). No significant differences were observed between axSpA patients and HCs in age (58.08 ± 9.83 vs 57.35 ± 10.54 years) or sex distribution. Audiometric assessment revealed a higher prevalence of hearing loss in axSpA patients than in HCs, affecting 46/53 patients versus 9/17 controls (86.8% vs 52.9%, p=0.006). This corresponded to an odds ratio of 5.84 (95% CI 1.69–20.20), a risk ratio of 1.64 (95% CI 1.03–2.60), and an absolute difference of 33.9 percentage points (95% CI 9.9–56.8). Regarding the audiological profile, hearing loss in the axSpA group was predominantly sensorineural, observed in 42/46 affected patients, while 4/46 presented mixed hearing loss; no cases of conductive hearing loss were identified. Hearing impairment was bilateral in all affected axSpA patients. In terms of severity, hearing loss was moderate in 13 patients, severe in 23, and profound in 10. Among HCs with hearing loss, the impairment was also predominantly sensorineural (8/9), with moderate, severe, and profound hearing loss observed in 4, 4, and 1 individuals, respectively. No significant differences in severity distribution were observed between groups (p=0.574), indicating that the between-group difference was mainly driven by a higher prevalence of hearing loss in axSpA patients rather than greater severity.

### Frequency-specific hearing threshold abnormalities in patients with axSpA

Pure-tone audiometry demonstrated consistently elevated air-conduction hearing thresholds in patients with axSpA compared with healthy controls across all evaluated frequencies (**Table 1**). Significant differences were observed at low and mid frequencies, including 0.25, 0.5, and 1 kHz, as well as at higher frequencies such as 6 and 8 kHz (all p<0.05). Although thresholds at 2 and 4 kHz were also numerically higher in the axSpA group, these differences did not reach statistical significance.

**Table 1:** Airway hearing thresholds in liminal tonal audiometry.

Frequency (KHz)	SpA hearing thresholds (dB)			HCs hearing thresholds (dB)			p
	RE (n=53)	LE (n=53)	Total (n=106)	RE (n=17)	LE (n=17)	Total (n=34)	
0.25	26.04 (12.14)	26.5 (11.42)	26.28 (10.03)	22.06 (6.39)	20.88 (3.64)	21.47 (4.15)	0.006*
0.5	27.26 (14.2)	25.94 (11.14)	26.6 (11.57)	21.76 (5.28)	20.88 (3.64)	21.32 (3.76)	0.005*
1	25.00 (13.44)	24.90 (10.4)	24.95 (11.1)	21.17 (3.32)	21.18 (3.32)	21.17 (2.81)	0.027*
2	28.02 (14.65)	26.79 (12.83)	27.40 (13.1)	23.53 (7.01)	24.12 (7.95)	23.82 (7.18)	0.287
4	39.05 (16.87)	35.75 (16.56)	37.4 (15.68)	29.12 (14.17)	30.29 (17)	29.70 (15.38)	0.081
6	43.86 (20.16)	41.32 (19.27)	42.59 (18.47)	32.65 (16.21)	31.76 (16.8)	32.20 (16.48)	0.036*
8	44.34 (22.66)	46.6 (20.84)	45.47 (20.57)	32.06 (17.95)	31.18 (17)	31.62 (17.27)	0.01*

Data are represented by mean (standard deviation). KHz, Kilohertz; dB, decibel; RE, right ear; LE, left ear. *Significant differences versus healthy controls. *p* < 0.05.

The greatest differences between groups were observed at high frequencies, particularly at 6 and 8 kHz, where patients with axSpA showed mean hearing thresholds above 40 dB, supporting the presence of clinically relevant sensorineural auditory impairment. However, increased thresholds at lower frequencies additionally suggest a more generalized auditory involvement rather than an exclusively high-frequency pattern.

### Association between hearing impairment, structural damage, persistent inflammation, and molecular profile

**Figure 1:**
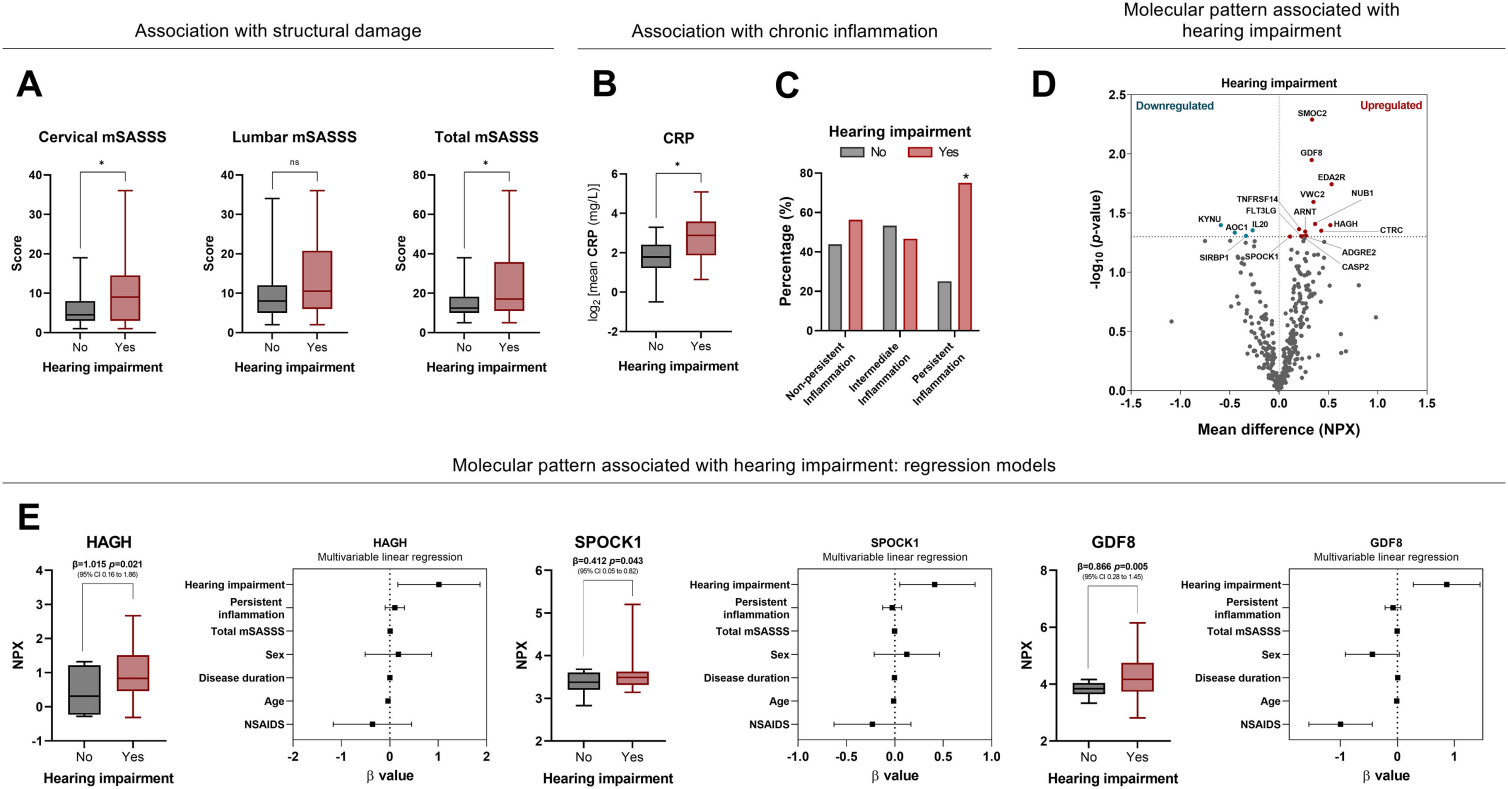
Clinical and proteomic features associated with hearing impairment in patients with axSpA. **(A)** Comparison of cervical, lumbar, and total mSASSS scores according to hearing impairment status. Patients with hearing impairment showed significantly higher cervical and total mSASSS scores. **(B)** Comparison of mean CRP levels over the previous 5 years between patients with and without hearing impairment. **(C)** Distribution of persistent inflammation categories according to hearing impairment status, showing a higher frequency of persistent inflammation among patients with hearing impairment. **(D)** Volcano plot illustrating the unadjusted differential expression analysis of circulating proteins associated with hearing impairment. The x-axis represents the mean difference in normalized protein expression (NPX) values, and the y-axis represents −log10(*p*-value). Proteins with higher expression in patients with hearing impairment are shown on the right, whereas proteins with lower expression are shown on the left. **(E)** Multivariable linear regression analyses evaluating the independent association between circulating protein levels and hearing impairment after adjustment for persistent inflammation status, total mSASSS, sex, disease duration, age, and NSAID use. Boxplots show NPX values according to hearing impairment status, and forest plots display β coefficients and 95% confidence intervals from the regression models. axSpA, axial spondyloarthritis; mSASSS, modified Stoke Ankylosing Spondylitis Spinal Score; CRP, C-reactive protein; NPX, normalized protein expression; NSAIDs, non-steroidal anti-inflammatory drugs; HAGH, hydroxyacylglutathione hydrolase; SPOCK1, SPARC/osteonectin, cwcv and kazal-like domains proteoglycan 1 (testican-1); GDF8, growth differentiation factor 8 (myostatin).

Patients with hearing impairment were older, had longer symptom duration, and showed worse ASAS-HI scores compared with those with normal hearing (**Table 2**), suggesting a greater cumulative disease burden and functional impact in patients with auditory impairment. No significant differences were observed regarding sex distribution, HLA-B27 positivity, disease activity indices, inflammatory markers or treatment exposure. Consistent with these findings, hearing impairment was also associated with markers of cumulative structural and inflammatory burden. Hearing impairment was associated with greater structural damage in patients with axSpA (**Figure 1A**). Patients with hearing impairment showed significantly higher cervical and total mSASSS scores compared with those without auditory involvement (*p* < 0.05), with a similar trend observed for lumbar mSASSS, suggesting a relationship between hearing dysfunction and cumulative structural burden. In addition, hearing impairment was associated with a higher inflammatory burden over time. Patients with hearing impairment showed significantly higher mean CRP levels during the previous 5 years compared with those without auditory involvement (**Figure 1B**), supporting the relationship between sustained systemic inflammation and hearing dysfunction. Furthermore, the proportion of patients with hearing impairment progressively increased across inflammation persistence groups, being highest in the persistent inflammation group compared with the intermediate and non-persistent inflammation groups (**Figure 1C**). To further characterize the molecular profile associated with hearing impairment, an initial unadjusted differential expression analysis was performed to identify proteins associated with hearing impairment (**Figure 1D**). To determine whether these associations were independent of relevant clinical factors, multivariable linear regression analyses adjusted for persistent inflammation status, total mSASSS, sex, disease duration, age, and NSAID use were subsequently performed (**Figure 1E**). Higher circulating levels of HAGH, GDF8, and SPOCK1 remained significantly associated with hearing impairment after adjustment for potential confounders. However, these associations did not remain significant after correction for multiple testing and should therefore be interpreted as exploratory.

**Table 2:** Clinical characteristics of axSpA patients according to the presence of hearing loss.

Clinical characteristic	Hearing impairment (n=46)	Normal hearing (n=7)	*p* value
Age (years)	60 (9.13)	47.86 (5.81)	0.001*
Sex (Male)	36 (78.3)	4 (57.1)	0.343
HLA B27	43 (95.6)	6 (100)	1
CRP (mg/L)	8.84 (11.69)	4.31 (3.4)	0.331
BASDAI	4.02 (2.61)	3.76 (2.85)	0.815
ASDAS	2.65 (1.2)	2.36 (0.8)	0.571
BASFI	36.57 (25.92)	32.87 (17.42)	0.932
ASAS HI	9.94 (4.33)	14.14 (3.7)	0.036*
Radiographic sacroiliitis	44 (93.6)	5 (71.4)	0.12
Disease duration (years) since symptom onset	35.73 (10.92)	24.71 (6.21)	0.012*
Treatments			
NSAIDs	40 (87)	4 (57.1)	0.086
Glucocorticoids	7 (15.2)	2 (28.6)	0.588

Data are represented by mean [standard deviation (SD)] for quantitative variables and total (percentage) for qualitative variables. axSpA, axial spondyloarthritis; CRP, C reactive protein (at the time of audiological evaluation); NSAIDs, Nonsteroidal Anti-inflammatory Drugs. *Significant differences among groups, *p* < 0.05.

## Discussion

In this cross-sectional study, patients with axSpA showed a markedly higher prevalence of hearing loss compared with matched healthy controls, predominantly bilateral sensorineural impairment. Hearing loss in axSpA was associated with older age, longer disease duration, greater structural damage, and sustained inflammatory burden, supporting the concept that auditory involvement may represent a chronic inflammation-related complication in axSpA.

Early reports attributed hearing impairment in ankylosing spondylitis primarily to conductive mechanisms related to inflammatory involvement of the middle ear and ossicular joints, supporting a mechanical origin of auditory dysfunction (14). Subsequently, studies suggested that both conductive and sensorineural mechanisms could coexist, reflecting possible cochleovestibular involvement beyond middle ear abnormalities (15, 16). More recent evidence has consistently identified sensorineural hearing loss as the predominant auditory pattern in axSpA, supporting the role of chronic inflammation, cochlear microvascular injury, and immune-mediated pathways in auditory dysfunction (4, 17). This shift in the understanding of auditory involvement in axSpA has progressively moved from a predominantly mechanical explanation toward the concept of chronic inflammation-driven cochlear and neurovascular damage.

Previous studies evaluating hearing impairment in axSpA have reported heterogeneous findings (5). Our results reinforce the association between axSpA and auditory dysfunction and suggest that hearing impairment may affect multiple frequency ranges. Consistent with our findings, Bulut Gökten et al. reported higher bone conduction hearing thresholds in patients with ankylosing spondylitis, supporting subclinical sensorineural auditory involvement associated with chronic disease burden (18). Although that study did not identify associations with conventional disease activity indices, our results suggest that sustained inflammatory burden over time, reflected by persistently elevated CRP levels, may be more closely related to auditory impairment than cross-sectional measures such as ASDAS. In one of the largest audiovestibular series to date, Amor-Dorado et al. evaluated 50 patients with AS and 44 matched controls, reporting a predominance of high-frequency sensorineural hearing loss together with vestibular involvement, including head-shaking nystagmus and abnormal caloric responses. Importantly, that study did not identify a significant association between audiometric findings and clinical or laboratory markers of inflammation (17). In contrast, our results link hearing impairment to cumulative inflammatory burden captured by serial CRP measurements over 5 years, reinforcing the notion that sustained systemic inflammation, rather than a single cross-sectional determination, may underlie auditory dysfunction in axSpA. Our study further extends these observations to the broader axSpA spectrum, including non-radiographic disease, and identifies a circulating proteomic signature not previously explored in this context.

To date, HAGH, SPOCK1, and GDF8 have not been previously associated with hearing impairment in axSpA, highlighting the exploratory nature of these findings. HAGH is involved in oxidative stress and inflammatory regulation through the glyoxalase system (19), whereas GDF8 participates in inflammatory signaling and bone remodeling pathways related to structural damage (20). In addition, SPOCK1 regulates extracellular matrix organization, tissue remodeling, and inflammatory responses (21). Therefore, these proteins may represent novel molecular markers associated with hearing impairment in axSpA, although their potential mechanistic role requires further investigation.

This study has some limitations that should be acknowledged, including its cross-sectional design and the relatively limited sample size, particularly in the healthy control group (n=17), which was smaller than the patient cohort; although the primary between-group comparison reached statistical significance, this imbalance may limit the precision of the patient–control comparisons, and the control estimates should be interpreted with caution. Therefore, causal relationships cannot be definitively established, and the proteomic findings should be interpreted as exploratory. Nevertheless, the integration of detailed audiological assessment with clinical and molecular analyses represents a major strength of the study and provides novel insights into the potential relationship between chronic inflammation and auditory dysfunction in axSpA. The high prevalence of threshold elevation observed in both patients and controls reflects the sensitive definition used: we defined hearing loss as a threshold >20 dB at any frequency. Although 20 dB corresponds to the lower limit of normal hearing established by the WHO (12), our per-frequency criterion is more sensitive than the WHO pure-tone average and captures early high-frequency changes that are common at the age of our cohort. Because this criterion was applied identically across groups, the significantly higher prevalence of hearing loss in patients supports a disease-related rather than a purely age-related effect.

A further consideration is that age-related hearing loss (presbycusis) is highly prevalent in this age range and may contribute to the auditory findings. Although controls were age- and sex-matched and evaluated with the same criterion, and age was included as a covariate in all multivariable models, residual confounding by presbycusis and other factors such as noise exposure or cardiovascular comorbidity cannot be fully excluded. The significantly higher prevalence and thresholds observed in patients compared with age-matched controls, however, argue against a purely age-related explanation.

In conclusion, hearing loss is highly prevalent in axSpA and appears to be associated with chronic inflammatory burden and structural damage. The proteomic findings suggest an exploratory circulating molecular profile associated with auditory involvement, which requires validation in larger longitudinal studies. From a clinical perspective, these results support considering early audiological assessment in patients with long-standing axSpA and structural damage to identify subclinical hearing impairment and optimize long-term patient management.

## Data Availability

The raw data supporting the conclusions of this article will be made available by the authors, without undue reservation.
